# Supplementary material for: Multilevel predictors of anaemia among pregnant women in Ghana: New evidence from the 2019 Ghana Malaria Indicator Survey
Source: PLOS Glob Public Health. 2024 Sep 5;4(9):e0003673. doi: 10.1371/journal.pgph.0003673 (PMC11376585; doi:10.1371/journal.pgph.0003673)
Supplement: Attachment — Submitted filename: Multilevel predictors of anaemia among pregnant women in Ghana.docx [file pgph.0003673.s001.docx]

**Multilevel predictors of anaemia among pregnant women in Ghana: New Evidence
from the 2019 Ghana Malaria Indicator Survey**

**Title**

The title is well-defined with much precision

**Abstract**

The section is well structured and written. However, ‘factors’ is erroneously written as ‘factrs’ at the results section. Authors are advised to carefully read the section and correct all punctuations as expected.

**Background**

It’s interesting to note how author brings to bear the common perceived need for this study. However, the problem statement is not clearly established; hence, authors are advised to clearly establish the claim for this study since a number of studies on the subject matter have been written in Ghana, with some focusing on a myriad of multilevel effects of the subject matter.

**Methods**

The section and analyses make the paper a well-grounded one and as a serious academic paper. The section is succinct and on point. However, matters and information on sampling and research design are not enough.

Authors are to stick to a particular writing style since both the British and American styles are combined in the manuscript ‘anaemia’ ‘sensitization.’ Either solely British or American is advised.

The last sentence of the Data collection sub-section uses ‘resting’ instead of ‘testing’ Authors are advised to make necessary correction.

**Discussion**

The discussion section is nicely structured; however, a few portions are poorly cited. In the first paragraph of the section, authors make claim on the comparison of anaemia prevalence in the study area and the global rate, where referenced item 19 is wrongly cited. Authors are advised to make amends.

**References**

The section is nicely carved out
