# Supplementary material for: Multilevel predictors of anaemia among pregnant women in Ghana: New evidence from the 2019 Ghana Malaria Indicator Survey
Source: PLOS Glob Public Health. 2024 Sep 5;4(9):e0003673. doi: 10.1371/journal.pgph.0003673 (PMC11376585; doi:10.1371/journal.pgph.0003673)
Supplement: Attachment — Submitted filename: Response to editor and reviewer comments.docx [file pgph.0003673.s002.docx]

**PLOS Global Health Journal**

Manuscript number: **PGPH-D-23-00541**

Manuscript title: **Multilevel predictors of anaemia among pregnant women in Ghana: New Evidence from the 2019 Ghana Malaria Indicator Survey**

**Point by point response to reviewer’s file**

| **Response to Editorial comments** | | |
| --- | --- | --- |
| **S/N** | **Comments** | **Response** |
| 1 | Please review your reference list to ensure that it is complete and correct. If you have cited papers that have been retracted, please include the rationale for doing so in the manuscript text, or remove these references and replace them with relevant current references. Any changes to the reference list should be mentioned in the rebuttal letter that accompanies your revised manuscript. If you need to cite a retracted article, indicate the article’s retracted status in the References list and also include a citation and full reference for the retraction notice. | Authors have reviewed the reference list and did not identify any retracted references. Again, all studies cited in the text are included in the reference list. Kindly refer to reference section on **pages 38 to 45.** |
| 2 | Please provide separate figure files in .tif or .eps format only and remove any figures embedded in your manuscript file. Please also ensure all files are under our size limit of 10MB. | Separate file has been provided for the figures and formatted in line with the journal guidelines. Kindly refer to files: **Fig 1 and Fig 2.** |
| 3 | We noticed you have some minor occurrence of overlapping text with the following previous publication(s), which needs to be addressed:  - <https://doi.org/10.1186/s12884-022-04586-2> - <http://dx.doi.org/10.1097/GRH.0000000000000063> - <https://doi.org/10.1186/s12887-023-03919-0>  In your revision ensure you cite all your sources (including your own works), and quote or rephrase any duplicated text outside the methods section. Further consideration is dependent on these concerns being addressed | These overlaps especially in the methods in reference to the underlisted studies have been corrected and rephased in the method section. Kindly refer to **pages 5-11.** |
| 4 | In the online submission form, you indicated that "Data will be made available upon request". All PLOS journals now require all data underlying the findings described in their manuscript to be freely available to other researchers, either 1. In a public repository, 2. Within the manuscript itself, or 3. Uploaded as supplementary information. This policy applies to all data except where public deposition would breach compliance with the protocol approved by your research ethics board. If your data cannot be made publicly available for ethical or legal reasons (e.g., public availability would compromise patient privacy), please explain your reasons by return email and your exemption request will be escalated to the editor for approval. Your exemption request will be handled independently and will not hold up the peer review process, but will need to be resolved should your manuscript be accepted for publication. One of the Editorial team will then be in touch if there are any issues. | This has been revised with the link to dataset provided. Kindly refer to **page 35.** |
| **Response to Reviewer comments** | | |
| **Abstract** | | |
|  | **Comments** | **Response** |
| 1 | The section is well structured and written. However, ‘factors’ is erroneously written as ‘factrs’ at the results section. Authors are advised to carefully read the section and correct all punctuations as expected. | Authors have revised this section. Kindly refer the result section of the abstract on **page 2.** |
| **Background** | | |
| 2 | It’s interesting to note how author brings to bear the common perceived need for this study. However, the problem statement is not clearly established; hence, authors are advised to clearly establish the claim for this study since a number of studies on the subject matter have been written in Ghana, with some focusing on a myriad of multilevel effects of the subject matter. | This section has been revised with the problem and knowledge gap clearly stated. Kindly refer to **page 5.** |
| **Methods** | | |
| 3.1 | The section and analyses make the paper a well-grounded one and as a serious academic paper. The section is succinct and on point. However, matters and information on sampling and research design are not enough. | The sampling and research design sub-sections has been revised and detailed information provided. Kindly refer to **pages 5 to 6.** |
| 3.2 | Authors are to stick to a particular writing style since both the British and American styles are combined in the manuscript ‘anaemia’ ‘sensitization.’ Either solely British or American is advised. | Authors have revised the manuscript and have adopted the British writing style. |
| 3.3 | The last sentence of the Data collection sub-section uses ‘resting’ instead of ‘testing’ Authors are advised to make necessary correction. | Authors have corrected the word “resting” to “testing”. Kindly refer to **page 35.** |
| **Discussion** | | |
| 4 | The discussion section is nicely structured; however, a few portions are poorly cited. In the first paragraph of the section, authors make claim on the comparison of anaemia prevalence in the study area and the global rate, where referenced item 19 is wrongly cited. Authors are advised to make amends. | This citation has been corrected. Kindly refer to **page 29.** |
